# Tau protein function: The mechanical exploration of axonal transport disorder caused by persistent pressure in dorsal root ganglia

**DOI:** 10.1002/mgg3.580

**Published:** 2019-01-29

**Authors:** Lei Zhang, Jun Fu, Xin‐Hua Cheng, Li Tang

**Affiliations:** ^1^ Department of Orthopedic Surgery Renmin Hospital Hubei University of Medicine Shiyan Hubei China; ^2^ Department of Anesthesiology Renmin Hospital Hubei University of Medicine Shiyan Hubei China; ^3^ Department of Microscopic Orthopaedic Renmin Hospital Hubei University of Medicine Shiyan Hubei China; ^4^ Department of Neurology Taihe Hospital Hubei University of Medicine Shiyan Hubei China

**Keywords:** axonal transport, neuron, phosphorylation, synapse, Tau

## Abstract

**Objective:**

We analyzed the function of Tau protein to explore the underlying mechanism of axonal transport disorder caused by persistent pressure in the dorsal root ganglia (DRG).

**Methods:**

Wistar rats were divided into the sham operated group, the control group and the experimental group. The Wistar rat model of continuous compression of DRG was used for further investigation. DRG neurons were extracted and cultured, and the protein content was detected using bicinchoninic acid method. Western blotting and immunofluorescence assays were performed to detect the protein content. Intraperitoneal injection of lithium chloride was performed for interaction with Tau. The results were then analyzed statistically.

**Results:**

After 2 weeks of sustained pressure, the expression level of Tau_396_ increased by 33%, while Tau_404_ increased by 25% in the DRG of the experimental group (*p* < 0.05). The expression level of PSD‐95 in the DRG decreased by 15% (*p* < 0.05), while the expression of vGluT1, vGluT3 and vAchT decreased significantly in the DRG of the experimental group (*p* < 0.05). There was no significant difference in the expression of vGluT2 and vGAT among the three groups (*p* > 0.05). After intervention with lithium chloride, the expression of phosphorylated Tau at the above sites decreased in varying degrees compared with the model group. The expression level of Tau_404_ was reduced by 55%, and that of Tau_199_ by 60% in the DRG of the experimental group.

**Conclusion:**

Chronic compression of DRG and hypoxia caused phosphorylation of Tau in axons and inhibition of PSD‐95, and the function of the synaptic glutamic acid vesicle is defective in the synapse. This process is crucial in the development and progression of axonal transport dysfunction induced by chronic DRG compression, and phosphorylation of Tau plays a substantial role in this process.

## 1 INTRODUCTION

Tau, a low molecular microtubule related protein, is concentrated in axons and dendrites, which are highly asymmetric phosphates. Tau binding is the core of early assembly of microtubules to promote assembly, stability, growth and development, and even transport of axonal microtubules, to maintain the distance between microtubules, which inhibits lipid peroxidation and tubulin aggregation (Alberts, Groen, Klein, Konieczny, & Koopman, 2014; Beharry et al., 2014). Several neurodegenerative diseases are known as Taupathies, which are characterized by the presence of straight or helical filamentous substances and highly phosphorylated Tau in neurons. The phosphorylation of Tau is the main means to regulate the function of neurons (Takenami, Hiruma, Kaneko, Okamoto, & Kawakami, 2015; Tang, Han, Liu, Li, & Wei, 2015). After abnormal phosphorylation, its misfolding (Tang et al., 2016) and molecular aggregation (Ahn et al., 2014) can weaken the function of its stable microtubules, resulting in the transport, storage and release of Tau (Frost, Gotz, & Feany, 2015), which leads to axonal transport disorder (Rodriguez‐Martin et al., 2016). Neuronal oxidative stress is an antecedent event of excessive phosphorylation of Tau, which leads to misfolding, high phosphorylation and abnormal aggregation of Tau, leading to axonal transport disorders. It has been reported that changes in the Tau phosphorylation can affect axon transport in the neurodegenerative disease model (Gao, Liu, Jiang, Ding, & Li, 2014; Wang, Wang, Li, Hao, & Wang, 2016). For example, the phosphorylation of the neurofilament and microtubule binding protein MAP1B increases the rate of their respective axon transport. In contrast, the increase of Tau phosphorylation resulted in an increase in the overall slow rate of Tau transport in neurons, and inhibited the phosphorylation of Tau by GSK‐3 and reduced its motility. The purpose of this study was to investigate the underlying mechanism of axonal transport disorder in the spinal DRG of rats through the study of Tau function.

## 2 MATERIALS AND METHODS

### 2.1 Material

#### 2.1.1 Experimental animal

Healthy adult male Wistar rats of similar age weighing between 220 and 260 g were purchased from the University Laboratory Animal Center, which was in accordance with grade 2 animal standard established by the national laboratory.

#### 2.1.2 Main equipment and reagents

The animal surgery equipment package was purchased from Shenzhen Rui Wode Life Science and Technology Co., Ltd.; UV spectrophotometer was purchased from Shanghai Jing Branch Co.; American Bole gel electrophoresis equipment was purchased from Beijing Jiapeng Tongchuang Technology Development Co., Ltd. Hettich centrifuge was purchased from Beijing speed up Equipment Maintenance Co., Ltd. Electric heated water bath was purchased from Shanghai Zhicheng Analytical Instruments Manufacturing Co., Ltd.

Chloral hydrate solution (10% w/v) was purchased from Shanghai Yu Duo Biotechnology Co., Ltd. Penicillin G sodium salt was purchased from Beijing Lanbolide Trading Company.

### 2.2 Methods

#### 2.2.1 Animal model of continuous compression of DRG in rats

Wistar rats were given free access to water and food. The ambient temperature was (25.0 ± 1.0)°C with a humidity of (60 ± 5)% and 12 hr photoperiod. Before surgery, the rats were fasted for 12 hr, not limited to drinking water. Five percent chloral hydrate (350 mg/kg) was used for anesthesia by intraperitoneal injection in rats, atropine 0.04 mg was also injected intramuscularly, until the normal reflex and corneal reflex disappeared. Then, start with rat lumbar and was depilated. The rats were placed in the prone position on the operating table. After sterilizing the skin and spreading the sterile towel sheet, the mid line and right incision was performed along the spinous process at L4 and L5; then the epidermis, fascia and muscle were cut off to the root of the transverse process, and the anterior wall of the intervertebral foramen was located at the rear. After exposing the right intervertebral foramen at L4‐L5, a “U” shaped stainless steel rod that was 4 mm long and 0.63 mm in diameter was inserted into the spinal canal, and advanced along the spinal canal to the central part of the spinal canal, applying sustained and stable compression against the DRG and adjacent nerve root. Then, the wound was washed repeatedly with normal saline, and the back muscle, myofascia and skin were sutured with 4‐0 silk threads. Penicillin G sodium‐400,000 units was injected intraperitoneally to prevent postoperative infection. At the end of the operation, we closely monitored the vital signs of the rats, maintained the ambient temperature, and prevented the occurrence of hypothermia. When the rats were awake and their vital signs recovered steadily, they were caged separately for breeding. The rats in the sham operated group were not instrumented with stainless steel wire; the rest of the operation was the same as in the experimental group, and the blank control group did not undergo any operation.

#### 2.2.2 Culture of DRG neurons

In the three groups, the spinal dorsal root ganglia at L4 and L5 were extracted and placed in the prechilled D‐H balance salt solution, respectively. After removing the spinal nerve root and the nerve root outer membrane with the ophthalmic scissors, the tissue specimens 1–3 mm^3^ in size were repeatedly rinsed and morselled. After 1‐hr digestion with 1 ml collagenase at 37°C, the tissues were further digested with 2 ml trypsin at 37°C for 1 hr. Fetal calf serum was added to terminate digestion, and the DRG tissue was repeatedly pipetted to be rendered into cell suspensions, which were placed in a l0 ml centrifuge tube with filter net filtration, and clarified by centrifugation at 1,200 rpm for 10 min. The supernatant was discarded after adding Pereoll current configuration separation liquid 2M1, repeatedly pipette mixed, and clarified by centrifugation at 1,200 rpm for 10 min after adding DMEM high glucose medium with 10% fetal bovine serum (FBS), 50 ng/ml of nerve growth factor and 1% penicillin, slight wind and percussion for cell suspension. The cell suspension was inoculated on the polylinecoated glass slide. The medium was replenished every 2 days, and the DRG neurons on day 6 were cultured for subsequent experiments.

#### 2.2.3 Sample preparation

DRG neuron cells were cultured in 100 ml culture flasks and incubated at a constant temperature for 24–36 hr. When the cells grew to a confluence of 70%–81%, 1.0 μmol/L nuclear inhibitor was added for 2 hr to extract protein.

#### 2.2.4 Main outcome measures

The protein extract was added at a ratio of 1:9 (i.e. 100 μg weight: 900 μl volume) and 10% protein homogenate was blown with a 1 ml homogenizer at 0°C in ice water. The homogenate was centrifuged at 1,200 rpm for 5 min, the supernatant was collected and the buffer solution was added, shaken on a shaker for 60 s and placed in boiling water for 3–5 min. The protein content in the sample was measured using the bicinchoninic acid method (BCA). Western blotting and immunofluorescence assays were performed.

#### 2.2.5 Postoperative drug intervention experiment

Once the model was established, 20 mg/kg lithium chloride solution was intraperitoneally injected once a day for 3 days. The control group was given an equivalent volume of normal saline immediately after successful modeling, once a day for three consecutive days. The DRG of L4 and L5 were harvested at the second week after continuous DRG compression and the DRG of L4 and L5 were also collected from the sham operation group and the normal control group, and the DRG specimens were placed in prechilled DH balanced salt solution. After the above operation, we performed Western blotting, and immunofluorescence assays.

### 2.3 Statistical analysis

Data were expressed as *M* ± *SD* ($\overset{¯}{x}\pm s$). The study used the United States IBM SPSS17.0 statistical software to analyze the differences between the test results of the first group using oneway analysis of variance. The LSD test was applied for comparison among groups if there was significant statistical difference. A *p *< 0.05 was defined as statistically significant.

## 3 RESULTS

### 3.1 Tau was significantly hyperphosphorylated in the DRG of the experimental group

**Figure 1:**
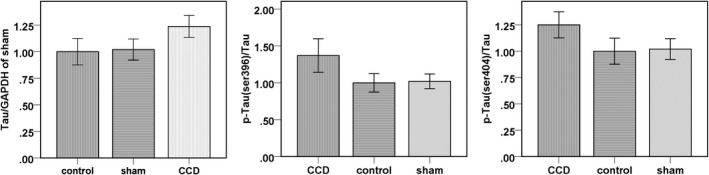
The level of phosphorylation of Tau protein in the DRG of the experimental group is significantly higher

The experimental results showed that the expression of Tau in the DRG of Wistar rats increased significantly after 2 weeks of continuous DRG compression, especially at Ser_396_ (Tau_396_) and Ser_404_ (Tau_404_). The above sites were exactly where Tau was located on the postsynaptic membrane. Compared with the normal control group and the sham operated group, Tau396 expression increased by 33% in the DRG of the experimental group after 2 weeks of continuous compression (the expression level of the Tau protein in the normal group was set as “1”) while Tau404 expression increased by 25% (*p* < 0.05). Compared with the normal control group and the sham operated group, the expression level of Tau396 in the DRG increased by 33% after 2 weeks of continuous compression, while the expression level of Tau404 increased by 25% (*p* < 0.05) (Figure 1).

### 3.2 Study on the expression of PSD‐95 n in the DRG of the experimental group

**Figure 2:**
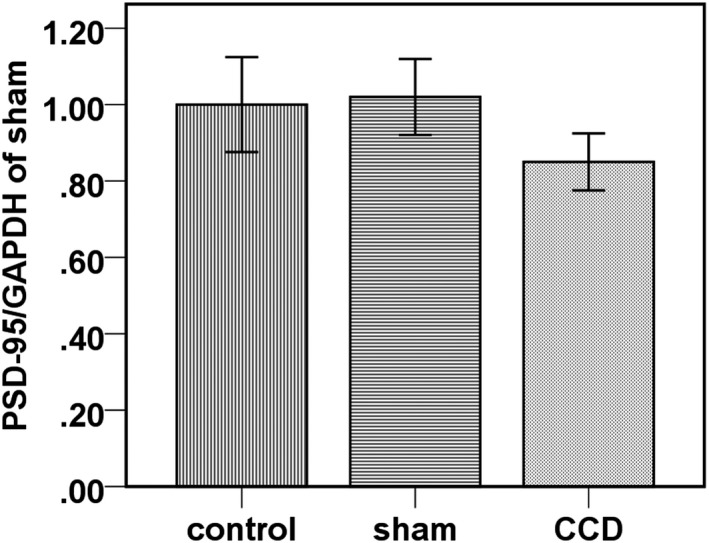
The level of PSD‐95 in the DRG is significantly decreased in the experimental group

Compared with the normal control group and the sham operated group, the expression level of PSD‐95 protein in the DRG of the experimental group was reduced by 15% (*p* < 0.05) after 2 weeks of continuous compression. Compared with the normal control group and the sham operated group, the expression of PSD‐95 decreased by 15% (*p* < 0.05) in the DRG rats after 2 weeks of continuous compression (Figure 2).

### 3.3 Western blotting assays of DRG in experimental group

**Figure 3:**
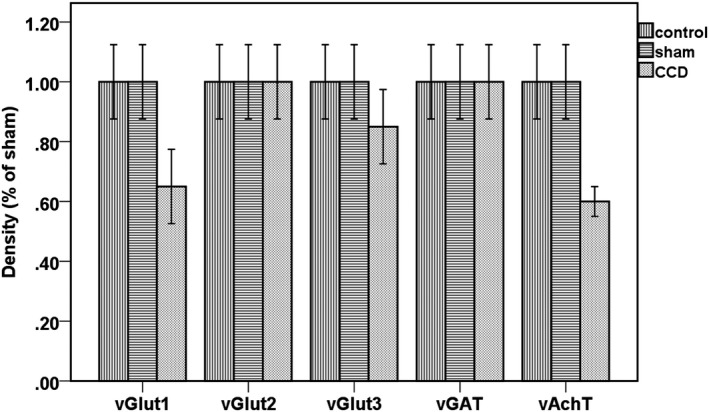
Western blotting of protein samples from DRG in the experimental group

Compared with the normal control group and sham operated group, the expression of vGluT1, vGluT3 and vAchT in DRG of the experimental group decreased significantly (*p* < 0.05) after 2 weeks of continuous compression. But no significant difference in the expression of vGluT2 and vGAT was observed between the normal group, the sham operated group and the experimental group (*p* > 0.05). Compared with the normal control group and the sham operated group, the expression of vGluT1, vGluT3 and vAchT in the DRG of rats in the experimental group decreased significantly after 2 weeks of sustained compression (*p* < 0.05) (Figure 3).

### 3.4 Immunohistochemically results of DRG in experimental group

**Figure 4:**
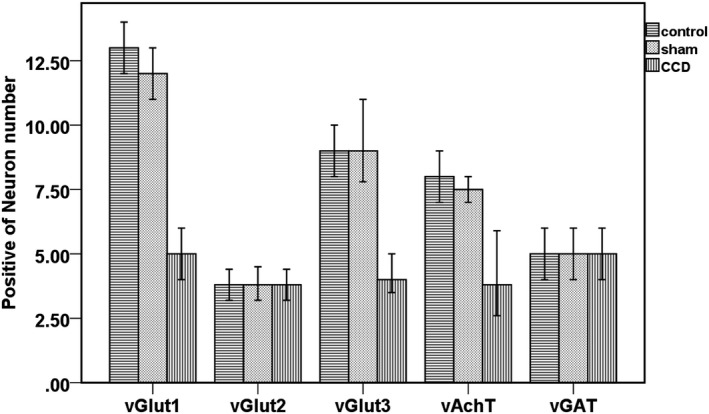
DRG immunohistochemical staining results in the experimental group

Compared with the normal control group and the sham operated group, the level of positive neurons of vGluT1, vGluT3 and vAchT in the DRG of experimental group decreased significantly after 2 weeks of continuous compression (*p* < 0.05). However, there was no significant difference between the positive neurons of vGluT2 and vGAT in normal group, sham operated group and experimental group (*p* > 0.05). Compared with the normal control group and the sham operated group, the level of positive neurons of vGluT1, vGluT3 and vAchT in the DRG of the experimental group decreased significantly 2 weeks after continuous compression (*p* < 0.05) (Figure 4).

### 3.5 The effect of lithium chloride on Tau phosphorylation

**Figure 5:**
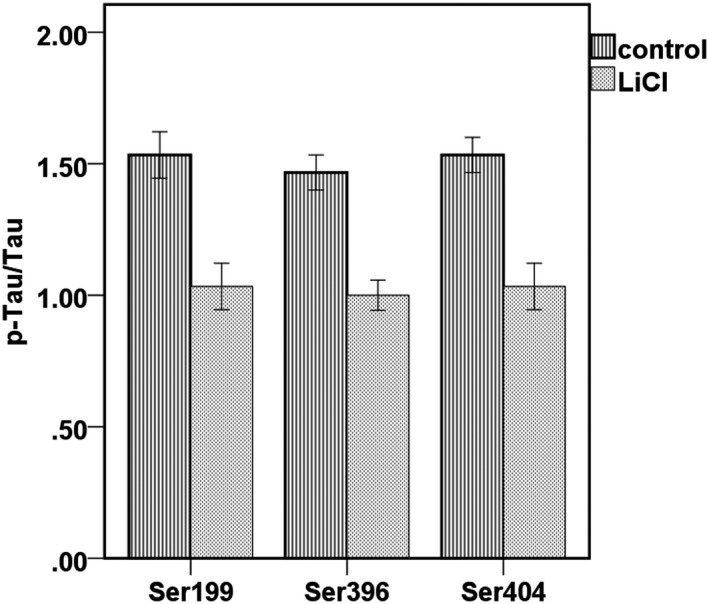
Effect of lithium chloride on Tau phosphorylation

The results showed that both continuous DRG compression and lithium chloride did not affect the overall expression of Tau after DRG compression (*p* < 0.05). However, Tau phosphorylation was significantly elevated at Ser_202_, Ser_404_, Ser_199_ and Ser_396_. Lithium chloride intervention reduced Tau phosphorylation at these sites to varying degrees compared with the model group. Tau_404_ decreased by about 55%, Tau_199_ by about 60% and Tau_396_ by about 50% in the DRG in the experimental group (*p* < 0.05). The above results show that lithium chloride can improve Tau phosphorylation level in the above sites in DRG neurons. Tau404 expression decreased by about 55%, Tau199 expression by about 60%, and Tau396 expression by about 50% in the DRG of rats in the experimental group (*p* < 0.05) (Figure 5).

## 4 DISCUSSION

The rat model of sustained compression of DRG is one of the ideal animal models to simulate degenerative changes of human spine into disc and ligament hypertrophy to spinal nerve roots (Ikeda et al., 2017) In this study, we successfully established a rat model of continuous DRG compression.

In pathological conditions, the phosphorylation of Tau protein as well as increased expression of Tau will accumulate to be active, which can reduce the binding of Tau protein and its stable microtubule function, resulting in the formation and repression of the microtubule assembly process. Therefore, the abnormal transport, storage and release process of neurotransmitters will occur and result in neurological damage (Takenami et al., 2015). PSD‐95 plays a substantially important role in the process. Although PSD‐95 is not active on its own, it can mediate the interaction between protein molecules and specifically binds to the NMDA receptor. NMDA receptor and synaptic membrane surface protein play an intermediary role in the synaptic level of nerve excitability and signal integration and conduction (Wang, Tan, & Yu, 2015). It has been reported (Scholz & Mandelkow, 2014) that knock‐out of *PSD95* in animals can induce longterm potentiation (LTP) induced disorders and learning and memory loss, indicating the significant role of PSD‐95 in the regulation of synaptic function as well as in the ability of neuronal cell survival. Our results showed that Tau phosphorylation, especially in Ser_396_ (Tau_396_) and Ser_404_ (Tau_404_), was significantly increased in DRG neurons after sustained compression of DRG in rats. The above sites were exactly where Tau was located on the postsynaptic membrane. Compared with the normal control group and the sham operated group, PSD‐95 expression in the DRG in experimental group decreased by 15% after 2 weeks of continuous compression (*p* < 0.05). Therefore, it is likely that PSD‐95 is involved in the regulation of synaptic function.

Studies (Lv et al., 2016; Wang, Hurwitz, et al., 2015) have shown that Tau is more sensitive in neuronal ischemic injury after molecular markers. In the vascular dementia rat model, Tau expression was significantly increased, and neuronal apoptosis occurred simultaneously, suggesting the function of Tau phosphorylation in initiating cellular apoptosis (Li, Chen, Chen, Cong, & Chen, 2016). In our study, we found that vGlut3, a glutamate transporter that is closely related to PSD‐95, was significantly decreased in the rat DRG after sustained compression of DRG. As an important excitatory neurotransmitter in rats, glutamate is closely related to synaptic function. Glutamic acid released by the body is mainly stored in synaptic vesicles after uptake by glutamate vesicles (Wang & Wang, 2015). To date, researchers have found three subtypes of vesicular vesicle transporters. Among them, vG1uT1 and vGluT2 are highly specific markers of glutamatergic neurons and are abundant in axon terminals. The results show that (Liem, van Dongen, Huygen, Staats, & Kramer, 2016; Sengupta, Bocchio, Bannerman, Sharp, & Capogna, 2017) the level of synaptic vesicle filling depends on the level of vesicular transporter expression, whereas the level of synaptic vesicle filling is ultimately the determining factor of synaptic vesicle size. Therefore, the amount of glutamate vesicle transporter vesicles and extracellular vesicle glutamate concentration determines the rate of glutamate uptake by the vesicles and the highest level of glutamate uptake, ultimately determining the intensity of synaptic transmission. In this study, we found that vGluT1, vGluT3 and vAchT significantly decreased in DRG neurons in the experimental group after 2 weeks of continuous compression (*p* < 0.05). There were no significant differences in the levels of vGluT2 and vGAT positive neurons (*p* > 0.05).

In conclusion, this study demonstrates that chronic DRG compression and hypoxia induced Tau phosphorylation in axons and inhibition of PSD‐95. The function of the synaptic glutamic acid vesicle is defective in the synapse. This process is crucial in the development and progression of axonal transport dysfunction induced by chronic DRG compression and hypoxia, and phosphorylation of Tau plays a crucial part in this process.

## CONFLICT OF INTEREST

The authors declare that there is no conflict of interest involved in this study.
